# 合并*MET*基因变异的非小细胞肺癌治疗研究进展

**DOI:** 10.3779/j.issn.1009-3419.2022.101.54

**Published:** 2022-12-20

**Authors:** 然 陈, 昌 江, 俊 唐, 丽 马, 树才 张

**Affiliations:** 1 441000 襄阳，湖北医药学院附属襄阳市第一人民医院肿瘤科 Department of Oncology, Xiangyang No.1 People's Hospital, Hubei University of Medicine, Xiangyang 441000, China; 2 101149 北京，首都医科大学附属北京胸科医院，北京市结核病胸部肿瘤研究所肿瘤内科 Department of Medical Oncology, Beijing Chest Hospital, Capital Medical University, Beijing Tuberculosis and Thoracic Tumor Research Institute, Beijing 101149, China; 3 330000 南昌，江西省肿瘤医院胸部肿瘤科 Department of Thoracic Oncology, Jiangxi Cancer Hospital, Nanchang 330000, China

**Keywords:** 肺肿瘤, *MET*扩增, 跳跃突变, MET酪氨酸激酶抑制剂, Lung neoplasms, *MET* amplification, Skipping mutations, MET-TKIs

## Abstract

间质-上皮细胞转化因子（mesenchymal-epithelial transition factor, *MET*）长期以来被认为是非小细胞肺癌（non-small cell lung cancer, NSCLC）发生发展过程中除表皮生长因子受体（epidermal growth factor receptor, *EGFR*）、间变性淋巴瘤激酶（anaplastic lymphoma kinase, *ALK*）、原癌基因酪氨酸蛋白激酶（c-ros oncogene 1 receptor tyrosine kinase, *ROS1*）之外最重要和最有前景的驱动基因。近年来，靶向MET的治疗药物不断被研发并应用于临床。首先是合并*MET*外显子14跳跃突变的NSCLC患者疗效得到进一步的提高。另外，晚期*EGFR*突变型NSCLC患者EGFR酪氨酸激酶受体抑制剂（EGFR tyrosine kinase inhibitors, EGFR-TKIs）耐药后继发*MET*扩增时，联合MET-TKIs和EGFR-TKIs的方案带来了明显的生存获益等诸多进展。本文综述了在不同情况下，NSCLC患者合并*MET*不同变异类型的治疗进展，为临床治疗策略的选择提供参考。

## Abstract (en)

Mesenchymal-epithelial transition factor (*MET*) has long been considered as the most crucial and promising driver gene in the occurrence and development of non-small cell lung cancer (NSCLC), except for epidermal growth factor receptor (*EGFR*), anaplastic lymphoma kinase (*ALK*), and *c-ROS* oncogene 1 receptor tyrosine kinase (*ROS1*). In recent years, therapeutic drugs targeting MET have been continuously developed and applied in clinical practice. First, the curative effect of NSCLC patients with *MET* exon 14 skipping mutations has been further improved. In addition, when *MET* amplification occurs after resistance to EGFR tyrosine kinase inhibitors (EGFR-TKIs) in patients with advanced *EGFR*-mutant NSCLC, the combination of MET-TKIs and EGFR-TKIs has brought significant survival benefits and many other advances. This article reviews the treatment progress of NSCLC patients with different types of *MET* variants under different circumstances, which provides reference for the selection of clinical treatment strategies.

## 1 前言

截至2021年，包括国家国立综合癌症网络（National Comprehensive Cancer Network, NCCN）、国际肺癌研究协会（The International Association for the Study of Lung Cancer, IASLC）、中国临床肿瘤学会（Chinese Society of Clinical Oncology, CSCO）、美国临床肿瘤学会（American Society of Clinical Oncology, ASCO）、欧洲医学肿瘤学会（European Society for Medical Oncology, ESMO）在内的多种权威临床指南建议对NSCLC进行超过10种基因变异的检测，其中包括：表皮生长因子受体（epidermal growth factor receptor, *EGFR*）、间变性淋巴瘤激酶（anaplastic lymphoma kinase, *ALK*）、原癌基因酪氨酸蛋白激酶（c-ros oncogene 1 receptor tyrosine kinase, *ROS1*）、间质上皮细胞转化因子（mesenchymal-epithelial transition factor, *MET*）外显子14跳跃突变及*MET*扩增、酪氨酸激酶受体编码基因（rearranged during transfection, RET）融合、鼠类肉瘤滤过性毒菌致癌同源体B（v-raf murine sarcoma viral oncogene homolog B, BRAF）、鼠类肉瘤病毒癌基因（Kirsten rat sarcoma viral oncogene, *KRAS*）、人类表皮生长因子受体2（human epidermal growth factor receptor 2, *HER2*）、神经营养因子受体络氨酸激酶（neurotrophin receptor kinase, *NTRK*）等。靶向其中7个基因的靶向药物已被美国食品药品监督管理局（Food and Drug Administration, FDA）批准并应用于临床。同样在国内，靶向*EGFR*、*ALK*、*ROS1*等的靶向药物也纷纷获批，明确推荐为晚期非小细胞肺癌（non-small cell lung cancer, NSCLC）合并相应基因突变的一线标准治疗^[1]^。继*EGFR*、*ALK*、*ROS1*之后，*MET*长期以来被认为是最重要和最有前景的驱动基因和治疗靶点^[2]^，受到广泛的关注。

*MET*原癌基因受体酪氨酸激酶和肝细胞生长因子（hepatocyte growth factor, HGF）受体同属于受体酪氨酸激酶（receptor tyrosine kinases, RTKs）家族。MET与其天然配体HGF形成HGF/MET轴，一起参与转导途径并调节正常生理条件下的基本细胞过程^[3]^。*MET*不同的致癌改变包括：*MET*突变、*MET*扩增、*MET*过表达、染色体重排和融合等，这些变异类型均可导致HGF/MET轴的失调，促成包括NSCLC在内广泛的人类癌症的发生发展^[4, 5]^。除了生理和病理作用以外，越来越多的证据^[6-8]^表明：由于MET与其他RTKs信号通路之间存在相互串扰作用，*MET*变异被发现是包括*EGFR*、血管内皮生长因子受体（vascular endothelial growth factor receptor, *VEGFR*）、*ALK*、*ROS1*、*RET*在内的多个靶点靶向药物的耐药机制。*MET*外显子14跳跃突变和*MET*扩增是*MET*多种变异形式中与生存预后显著相关的变异形式。合并此类*MET*变异的肿瘤患者更容易发生远处转移，被认为是NSCLC明确的预后不良因素，因而促进了多项相关临床研究^[9-11]^的开展及相应靶点靶向药物的研发。近来有不少临床及临床前研究显示：带有*MET*外显子14跳跃突变的肿瘤细胞大多对MET-TKIs治疗敏感，所以与历史数据相比，在靶向MET的药物出现以后，*MET*外显子14跳跃突变的NSCLC患者预后变得更好。

EGFR-TKIs的耐药机制问题一直是肿瘤临床中面临的热点和难点问题。近年来*MET*扩增被发现是晚期*EGFR*突变NSCLC患者EGFR-TKIs的明确耐药机制，并且有临床试验^[12-14]^显示：以MET-TKIs为基础联合EGFR-TKIs的用药方案给这部分人群带来了显著的临床获益，从而成为部分EGFR-TKIs耐药后患者的潜在有效选择。

在过去的十余年中，面对合并*MET*变异的肿瘤，多种MET抑制剂、单克隆抗体、双特异性抗体、抗体药物偶联药物、小分子制剂已经被研发出来，并处于不同的临床评估阶段^[4, 5, 15]^。

2018年以来，随着免疫检查点抑制剂（immune checkpoint inhibitors, ICIs）在实体瘤中的广泛应用并获得突破性成果，ICIs在*MET*变异的NSCLC中疗效的探索也逐渐受到大家的关注。

2021年6月，拥有我国自主知识产权的创新靶向药赛沃替尼正式获批上市，成为首个且唯一获批的MET抑制剂，用于携带*MET*外显子14跳跃突变的局部晚期或转移性NSCLC。除赛沃替尼以外，多种其他MET抑制剂在*MET*外显子14跳跃突变的晚期NSCLC患者中展现出良好的效果^[16-18]^。包括CSCO、中国非小细胞肺癌分子病理检测临床指南、NCCN等均已将*MET*外显子14跳跃突变检测纳入晚期NSCLC的一级或二级推荐。另外，MET蛋白过表达的检测近年来在多项临床试验中也展现出潜在的应用价值而逐渐受到业界的重视^[12, 14, 19-22]^，并在2022版中华医学会肺癌临床诊疗指南中被推荐。

## 2 *MET*主要变异类型的检测现状

### 2.1 *MET*外显子14跳跃突变的检测

目前*MET*外显子14跳跃突变的主要检测手段为第二代测序（next-generation sequencing, NGS）和定量逆转录聚合酶链反应（quantitative reverse transcription polymerase chain reaction, RT-qPCR）。二者适用于不同的样本类型，技术特点不同，各有优劣。

#### 2.1.1 NGS检测

##### 2.1.1.1 DNA的NGS测序

以DNA为检测对象，用扩增子法或杂交捕获法建库富集*MET*外显子14跳跃相关区域片段进行基因测序检测。测序的适用样本类型优选肿瘤组织学样本或细胞学样本。此类检测测序的优点为：通量高，准确性亦较高。缺点为：检出率受引物设计或探针覆盖及生物信息分析能力的影响、检测周期相对较长。此方法在临床上推荐程度较高。若检测样本为液体活检样本，因循环肿瘤DNA（circulateing tumor DNA, ctDNA）在患者体内含量较低，对检测方法的灵敏度要求较高，可能出现假阴性，并且ctDNA的检测同样会受到探针覆盖的影响。在使用此类标本进行检测时因充分考虑到局限性，所以尽可能避免使用此类标本进行检测，推荐程度一般。如若在DNA测序中检测到疑似*MET*外显子14跳跃突变时，建议辅以RT-qPCR或RNA测序来进一步明确。

##### 2.1.1.2 RNA的NGS测序

在RNA水平上检测*MET*外显子13/15融合来判断是否发生*MET*外显子14跳跃突变。测序的适用样本类型可为肿瘤组织学样本或细胞学样本。该方法直接检测*MET*外显子14跳跃突变，检测覆盖范围明确，生信分析简单。当发生影响剪切的非典型内含子突变时，有助于识别*MET*外显子14跳突。但对一些*MET*的罕见突变可能出现漏检。RNA测序优点是通量高、准确性高。缺点是对样本质量要求高、检测全程质控要求严格、检测周期长、可及性相对较低。此检测方法在临床上推荐程度高。

#### 2.1.2 RT-qPCR检测

以RNA为检测对象，在mRNA水平逆转录后，用荧光PCR检测*MET*外显子13/15融合。在*MET*外显子13/15之间的区域设计引物，检测是否存在特异表达的扩增产物，此方法拥有较高的检测准确率^[23]^，但对*MET*外显子14跳跃功能相似的*MET*罕见突变易发生漏检，如Y1003位点氨基酸改变或缺失^[24, 25]^。该方法的检测样本类型可为组织学或细胞学样本，优点是准确性高、平台可及性高、周期较短。缺点则是RNA标本易降解，对样本治疗要求高。此方法在临床中的推荐程度亦较高。

### 2.2 *MET*基因扩增的检测

*MET*基因扩增的检测方法主要包括荧光原位杂交（fluorescence *in situ* hybridization, FISH）和NGS。另外，近年来还出现了*MET*扩增检测的新策略，目前尚处于探索阶段。需要我们注意的是：目前判断*MET*基因扩增的标准和临床获益阈值并非一种“全或无”的状态，在国内和国际上尚没有固定统一的标准，所以在判定*MET*扩增时需要参照相应的临床研究并结合临床实际情况进行综合考虑。

#### 2.2.1 FISH检测

该技术是检测*MET*基因扩增的金标准。通过荧光探针原位标记*MET*基因，观察肿瘤细胞中MET荧光信号的数量来计算肿瘤细胞中*MET*基因拷贝数（gene copy number, GCN），或通过标记*MET*基因和第7号染色体着丝粒（CEP7），计算肿瘤细胞中MET/CEP7比值。临床中通过*MET*基因GCN和MET/CEP7比值来判断扩增。目前FISH检测判断*MET*扩增的标准主要参照的是UCCC标准和Cappuzzo标准，推荐阳性阈值为*MET*基因GCN > 5或MET/CEP7比值≥2，当出现GCN > 5且MET/CEP7比值< 2时应考虑为多倍体。该检测技术适用的样本类型为组织学及细胞学样本。优点是基因扩增检测的金标准、可区分多倍体和局部扩增、检测中所需的样本也较少。缺点是单基因检测、缺乏绝对统一的判断标准。此方法在临床中推荐程度极高。

#### 2.2.2 DNA二代测序

基于测序深度和特定位点变异频率等对*MET*基因GCN进行计算，适用样本首选组织学样本或细胞学样本。目前不同检测平台使用的NGS测序pannel和生信分析策略也不尽相同，检测结果呈现形式各异。面对液体活检样本进行NGS判别*MET*扩增仍存在巨大挑战，需优先采用组织样本进行检测，若出现液体样本检测阴性时并不能排除扩增阳性。NGS检测同时还受肿瘤细胞百分比和生物信息算法影响，成熟度不高有待验证。在临床中推荐程度偏低。

#### 2.2.3 关于*MET*扩增新策略的探索

数字微滴PCR（droplet digital PCR, ddPCR）是一种新技术，对微滴体系进行扩增反应后，分析每个微滴的荧光信号，将判断结果按泊松分布，读取靶标和内参核酸的阳性微滴个数及比例从而得到靶分子拷贝数及浓度。目前在检测*MET*扩增，特别是在液体活检标本中已有相关探索^[26, 27]^。

### 2.3 MET蛋白过表达的检测

MET蛋白过表达的检测方法为免疫组织化学法（immunohistochemistry, IHC），利用抗原抗体特异性结合，通过化学反应标记抗体的显色剂显色来判定肿瘤组织中MET蛋白表达情况。目前国内外涉及检测MET蛋白过表达的抗体克隆号繁多，不同抗体的染色型存在差异，仍没有统一的判读标准。目前相关的临床研究判读标准结合相关抗体在组织中的表达百分比及表达强度进行综合判断，例如TATTON研究和SAVANNAH研究中入组的为MET IHC≥50%、肿瘤细胞强染色（3+）的患者，抗体克隆号为SP44^[14]^，INSIGHT研究入组的为MET IHC≥50%、肿瘤细胞强染色（3+）或中等染色（2+）的患者，抗体克隆号为D1C1^[12]^等。鉴于当前检测MET蛋白过表达的抗体多样性及判读标准的不确定性，对不同抗体的一致性进行对比十分重要并需要更多的临床试验来证实MET蛋白过表达的临床意义。

## 3 合并*MET*基因主要变异形式的NSCLC治疗进展

### 3.1 *MET*外显子14跳跃突变NSCLC的治疗

*MET*外显子14跳跃突变被认为是NSCLC中一个独立的驱动突变，通常与其他驱动突变（如*EGFR*、*ALK*、*ROS1*）相互排斥，与不良预后相关^[28]^。Awad等^[29]^和Tong等^[9]^研究显示：*MET*外显子14跳跃突变代表了NSCLC的一种独特的临床分子亚型，有助于对患者进行个性化治疗的分层。近年来，随着NGS检测在恶性肿瘤分子诊断中的应用和普及，*MET*变异也更多地被检测出来，促进了MET靶向治疗的发展。包括MET-TKIs、ICIs、抗体偶联药物、单克隆抗体、双特异性抗体等在内的许多相关治疗进展正在被报道，其中有关于*MET*外显子14跳跃突变的TKIs治疗相关的临床研究数据汇总见表 1。

**表 1:** *MET*外显子14跳跃突变患者接受MET-TKIs治疗的临床研究 Clinical trails including *MET* exon 14 skipping mutant NSCLC treated with MET-TKIs

Researcher	Trial Identifier/ Treatment method/ Phase/ Expiration date	Trail design (endpoints, number of patients)	Outcomes (ORR, mDOR, mPFS, mOS, TTR)
Drilon *et al*^[30, 31]^	PROFILE 1001 Crizotinib Phase I 2020-07	ORR, mDOR, mPFS, mOS, *n*=69 *MET* exon 14	ORR: 32%; mDOR: 9.1 mon; mPFS: 7.3 mon; mOS: 20.5 mon
Paik *et al*^[32, 33]^	VISION Tepotinib Phase II 2021-12	ORR, mDOR, mPFS, mOS, *n*=146 *MET* exon 14	ORR (by IRC): 44.7%; ORR (by investigator): 56%; mDOR: 11.1 mon; mPFS: 8.5 mon; mOS: 17.1 mon
Wolf *et al*^[18, 33]^	GEOMETRY mono-1 Capmatinib Phase II 2022-02	ORR, mDOR, mPFS, *n*=160 *MET* exon 14 (pre-treated=100, naive=60)	ORR (pretreated: 44%; naive: 66.7%); mDOR (pretreated: 9.7 mon; naive: 12.6 mon); mPFS (pre-treated: 5.5 mon; naive: 12.3 mon)
Lu *et al*^[16]^	NCT02897479 Savotinib Phase II 2021-12	ORR, mDOR, TTR, mPFS, mOS *n*=70 *MET* exon 14 (25 sarcomatoid carcinoma)	ORR: 49.2%; mDOR: 8.3 mon; TTR: 1.4 mon; mPFS: 6.8 mon; mOS: 12.5 mon
NSCLC: non-small cell lung cancer; MET-TKIs: mesenchymal epithelial transition factor tyrosine kinase inhibitors; IRC: Independent Review Committee; ORR: objective response rate; mPFS: median progression-free survival; mOS: median overall survival; mDOR: median duration of response; TTR: time to response.			

#### 3.1.1 克唑替尼单药治疗

克唑替尼是一种多靶点小分子TKIs，主要靶向*ALK*、*ROS1*和*MET*。克唑替尼对合并*MET*外显子14跳跃突变或*MET*扩增肿瘤中的疗效尚未在大规模人群中报道，目前有部分小规模的临床研究结果让我们看到了希望。Drilon等^[30]^在I期PROFILE 1001研究中（*n*=69）报道了*MET*外显子14跳跃突变的晚期NSCLC患者（其中38%为初始治疗）中单药克唑替尼治疗的疗效：客观缓解率（objective response rate, ORR）为32%（95%CI: 21%-45%）；中位无进展生存期（median progression-free survival, mPFS）为7.3个月（95%CI: 5.4-9.1）；中位缓解持续时间（median duration of response, mDOR）为9.1个月（95%CI: 6.4-12.7）；中位总生存时间（median overall survival, mOS）为20.5个月（95%CI: 14.3-21.8）。基于PROFILE1001的研究结论，克唑替尼于2018年5月成为首个获得FDA突破性治疗认定的MET-TKIs^[31]^，用于先前接受过含铂药物化疗，含*MET*外显子14跳跃突变的转移性NSCLC患者的治疗。克唑替尼在特泊替尼和卡马替尼未获批或未上市的情况下使用较为普遍。

#### 3.1.2 特泊替尼单药治疗

特泊替尼是一种选择性MET抑制剂，通过破坏MET信号转导途径，从而显示出潜在的抗肿瘤活性^[32]^。VISION研究^[33]^是由Paik等开展的一项II期试验，该研究评估了特泊替尼500 mg每日一次对152例*MET*外显子14跳跃突变的NSCLC患者（包括经治和初治患者）的临床活性，其中有99例患者随访时间达9个月以上。研究结果显示：近一半口服特泊替尼的患者与部分缓解相关，独立评审委员会（Independent Review Committee, IRC）评审的ORR为44.7%（95%CI: 36.7-50），研究者评估的ORR为56%（95%CI: 45-66）。mDOR为11.1个月（95%CI: 8.4-18.5），mPFS为8.9个月（95%CI: 8.3-11.2）。初治和经治患者预后无统计学差异，初治患者ORR为44.9%（95%CI: 32.9-57.4），mPFS为8.5个月（95%CI: 6.8-11.3）。经治患者ORR为44.6%（95%CI: 33.7-55.9），mPFS为10.9个月（95%CI: 8.2-12.7）。基于VISION研究结果，日本厚生劳动省于2020年3月批准特泊替尼用于合并*MET*外显子14跳跃突变的不可切除、晚期或复发性NSCLC的治疗。

#### 3.1.3 卡马替尼单药治疗

卡马替尼也是一种高选择性MET的小分子抑制剂，通过阻断MET磷酸化^[10]^来阻止MET信号通路下游效应子的激活。Wolf等^[18]^进行了一项II期的多臂临床研究（GEOMETRY mono-1），研究中涵盖有已经接受一线或二线及以后治疗的*MET*外显子14跳跃突变的NSCLC患者（*n*=160）。此研究采用卡马替尼400 mg口服、每日两次的治疗方案，结果显示：经治患者人群ORR为44%（95%CI: 34%-54%），初治患者人群ORR为66.7%（95%CI: 53%-78%）。经治和初治患者人群的mDOR分别为9.7个月和12.6个月，mPFS分别为5.5个月（95%CI: 4.2-8.1）和12.3个月（95%CI: 8.2-21.6），mOS分别为13.6个月（95%CI: 8.2-22.2）和20.8个月（95%CI: 12.4-NR）。在安全性方面：卡马替尼和特泊替尼的安全性相似，两项试验中最常见的治疗相关不良反应（treatment-related adverse effects, TRAEs）是外周水肿和恶心。在特泊替尼试验中，治疗相关TRAEs导致33%的患者降低剂量，11%的患者永久停药，在卡马替尼试验中，这两项数据分别为23%和11%。在停药或减量后，TRAEs得到有效恢复^[18, 33]^。基于此研究结果，鉴于卡马替尼的有效性和安全性，于2020年5月FDA批准卡马替尼用于一线或先前接受过治疗的局部晚期或转移性*MET*外显子14跳跃突变的成人NSCLC患者的治疗。

#### 3.1.4 赛沃替尼单药治疗

赛沃替尼是一种高选择性、强效的MET受体抑制剂，通过三磷酸腺苷竞争方式干扰MET信号转导通路，抑制MET的激活，从而抑制肿瘤中的细胞生长^[34]^。Lu等^[16]^在中国的32家医院进行了一项多中心、单臂、开放性II期临床试验，评估了口服赛沃替尼400 mg和600 mg剂量每日一次，在合并*MET*外显子14跳跃突变的中国NSCLC患者中的疗效和安全性（*n*=70）。其中包含25例肺肉瘤样癌（pulmonary sarcomatoid carcinoma，PSC）患者和45例其他组织学类型的NSCLC患者，中位随访时间为17.6个月。主要终点为IRC评估的可评价集中患者的ORR。在全分析集中进行敏感性分析和安全性评估。在合并*MET*外显子14跳跃突变的NSCLC患者中，赛沃替尼显示出令人鼓舞的ORR结果。在可评价集中[*n*=61；ORR：49.2%；95%CI：36.1-62.3；响应时间（time to response, TTR）为1.4个月；DCR为93.4%；mDOR为8.3个月]，在全分析集中（*n*=70；ORR：42.9%，95%CI：31.1-55.3；DOR为8.3个月；PFS为6.8个月；mOS为12.5个月）。并且，在不同的组织病理学类型中ORR结果类似：在PSC患者人群中ORR为40%，mPFS为5.5个月；在其他病理类型的NSCLC中ORR为44.4%，mPFS为8.3个月。在初治和经治人群中的ORR结果也相似（经治患者中ORR为40.5%，在初治患者中ORR为46.4%）。事后分析发现：赛沃替尼对颅内病灶也能有效控制^[16]^，亚组分析提示：其中15例基线合并脑转移的患者ORR为46.7%，DCR高达93.3%。总的来说，*MET*外显子14跳跃突变定义了NSCLC的一种特殊基因组亚型，也证明了选择性MET-TKIs可能比多靶点、非选择性TKIs能提供更好的疗效。2020年7月，中国国家药品监督管理局授予赛沃替尼新药申请优先审查地位，并于2021年6月获批用于治疗*MET*外显子14跳跃突变的局部晚期或转移性NSCLC的治疗。

#### 3.1.5 免疫治疗在*MET*外显子14跳跃突变驱动的NSCLC中的作用

随着ICIs在NSCLC患者中获得成功，发现合并不同驱动基因分子亚型的NSCLC患者接受抗程序性死亡受体1（programmed cell death 1, PD-1）/程序性死亡配体1（programmed cell death ligand 1, PD-L1）抑制剂治疗疗效存在高度的异质性。*EGFR*、*ALK*、*ROS1*和*RET*相关的基因变异患者似乎无法从免疫治疗中获益。相反，*KRAS*或*BRAF*突变型肺癌患者使用ICIs可获得相对较高的应答率和长期受益^[35, 36]^。与其他突变驱动的肺癌相比，*MET*外显子14跳跃突变的肺癌表达更高水平的PD-L1，肿瘤突变负荷较低^[37]^。然而ICIs在*MET*外显子14跳跃突变NSCLC患者中的作用尚未明确。在一项临床研究^[37]^中147例*MET*外显子14跳跃突变NSCLC患者中，其中一组有24例患者接受ICIs治疗，其中11例为一线治疗，6例二线治疗，7例三线治疗。结果观察到的总体的反应率为17%（95%CI: 6%-36%），mPFS为1.9个月（95%CI: 1.7-2.7），mOS为18.2个月（95%CI: 12.9-NR）。在另一组接受ICIs治疗的46例患者中，*MET*外显子14跳跃突变的NSCLC患者接受免疫治疗的疗效与之前接受MET-TKIs治疗的情况无显著差异^[38]^。在另一方面，来自免疫靶标国际注册机构（Immunotarget International Registry, IIR）的23例接受ICIs治疗的*MET*外显子14跳跃突变的NSCLC患者中，该组的mOS为25个月（95%CI: 18.4-NR），明显长于他基因变异的分子亚型^[35]^。另有数据^[39]^报道：包含13例*MET*外显子14跳跃突变的NSCLC患者，经ICIs治疗后结果显示：其中6例（46%）患者的PFS为18个月-49个月。在MET-TKIs联合ICIs治疗的探索中，一项II期临床研究对卡马替尼和纳武利尤单抗联合使用的有效性和安全性进行了研究^[40]^，其中包括既往治疗过的*MET*外显子14跳跃突变的NSCLC患者。结果显示：ORR为25%，mPFS为13.8个月（95%CI: 3.5-19.2），有24%的患者报告有严重不良反应。

#### 3.1.6 单克隆抗体、抗体-药物偶联药物等新药

近来，有包括针对SEMA结构域（构成成熟MET蛋白的其中一个结构域）的单克隆抗体和靶向MET的抗体-药物偶联药物正在研发中。其中，Sym015^[41]^是一种针对MET非重叠表位的两个单克隆抗体的组合体，它在*MET*外显子14跳跃突变（*n*=12）和*MET*扩增（*n*=8）的NSCLC患者（包括初治和既往接受过MET-TKIs治疗过的患者）中进行了研究和探索。研究结果显示：*MET*外显子14跳跃突变初治队列的ORR为100%，中位DOR为6.5个月，中位PFS为9.2个月，而在既往接受过治疗的人群中有效率较低^[42]^。埃万妥单抗是全球首款针对EGFR和MET受体的双靶点特异性IgG1抗体，通过阻断*EGFR*突变体和c-MET通道、激活抗体依赖性细胞介导的细胞毒作用（antibody-dependent cell-mediated cytotoxicity, ADCC）和抗体依赖性细胞吞噬作用（antibody-dependent cellular phagocytosis, ADCP）从而发挥抗肿瘤作用，于2021年5月21日被FDA批准用于*EGFR*外显子20插入突变NSCLC患者的治疗。世界肺癌大会（World Congress of Lung Cancer, WCLC）2021也公布了Chrysalis研究^[43]^其中一个队列的初步结果，该队列探索了埃万妥单抗在19例*MET*外显子14跳跃突变的NSCLC患者中的疗效。结果发现：在14例疗效可评估患者人群中，ORR高达64%，9例应答患者中有8例依然持续反应。

### 3.2 EGFR-TKIs耐药后合并*MET*扩增/过表达的NSCLC的治疗

在*EGFR*突变的NSCLC中，由于MET和RTK（EGFR）信号通路之间存在串扰，MET的激活会对EGFR-TKIs的有效性产生负面影响^[44, 45]^。*MET*扩增通过持续激活EGFR下游的信号通路（如MAPK、STAT和PI3K/AKT介导的信号通路）导致耐药，这些独立于EGFR的激活和信号转导，属于旁路激活。在EGFR-TKIs耐药机制中，*MET*扩增是最为常见和重要的因素之一。一些临床前研究^[46, 47]^表明：在奥希替尼耐药后发生*MET*扩增的*EGFR*突变型NSCLC细胞系中，MET抑制剂与奥希替尼联用可明显克服奥希替尼耐药。也有一些临床前的模型使用高选择性MET抑制剂特泊替尼联合EGFR-TKIs（厄洛替尼、吉非替尼、阿法替尼等），最终看到：EGFR-TKIs耐药后出现*MET*扩增（包括高水平扩增和过表达）的肿瘤出现了明显缩小甚至完全消退^[48]^。在临床研究中：第一、二代EGFR-TKIs耐药后发生*MET*扩增的概率为10%-21%^[49, 50]^，第三代EGFR-TKIs耐药后发生*MET*扩增的概率为15%左右^[51, 52]^，并且在FLAURA研究中得以证实。所以，*MET*扩增也是最常见的获得性耐药机制（15%）^[51]^。此类患者接受传统化疗^[12]^或单一的MET-TKIs治疗等^[53]^方案效果均不理想。而以MET-TKIs为基础，联合EGFR-TKIs的双重抑制方案，相对于传统方案，在多项临床研究^[12-14]^中表现出明显的疗效。其中有关于*MET*扩增/*MET*过表达的TKIs治疗相关的临床研究数据汇总见表 2。

**表 2:** *MET*扩增/*MET*过表达患者接受MET-TKIs治疗的临床研究 Clinical trails including *MET* amplification/overexpression NSCLC treated with MET-TKIs

Researcher	Trial Identifier/ Treatment method/ Phase/ Expiration date	Trail design (endpoints, number of patient)	Outcomes (ORR, mDOR, mPFS, mOS)
Wu *et al*^[12]^	INSIGHT Tepotinib+Gefitinib Phase Ib/II 2017-05	mPFS, mOS *n*=19 *MET* amplification *n*=34 *MET* overexpression	*MET* amplification (mPFS: 16.6 mon; mOS: 37.3 mon) *MET* overexpression (mPFS: 8.3 mon; mOS: 37.3 mon)
Smit *et al*^[54]^	INSIGHT2 Tepotinib+Osimertinib Phase II 2022-04	ORR，mDoR *n*=139 *MET* amplification by FISH	ORR (follow-up time≥9 mon): 54.5% ORR (follow-up time≥3 mon): 45.8% mDOR: NR
Yu et al^[55]^	ORCHARD Savolitinib+Osimertinib Phase II 2021-01	ORR *n*=17 *MET* amplification	ORR: 41% (*n*=7, 95%CI: 25%-59%）
Oxnard *et al*^[56]^	TATTON Savolitinib+Osimertinib Phase Ib 2018-02	ORR *n*=36 *MET* amplification	ORR: 44% (*n*=15, 95%CI: 22%-69%)
Wu *et al*^[19]^	NCT01610336 Capmatinib+Gefitinib Phase Ib/II 2018-07	ORR *n* (Phase Ib)=61 *MET* amplification; *n* (Phase II)=100 *MET* amplification	ORR (Phase I/ II): 27% ORR (Phase II, MET GCN≥6): 47%
Fang *et al*^[57]^	NCT02374645 Savolitinib+Gefitinib Phase Ib 2020-10	ORR *n*=51 *MET* amplification	ORR (T790M negative): 52% (12/23) ORR (T790M positive): 9% (2/23) ORR (T790M unknown): 40% (2/5)

#### 3.2.1 特泊替尼+吉非替尼

INSIGHT研究是由Wu等^[12]^开展的一项Ib期/II期多中心随机临床试验，明确证实了这种双重抑制方案的有效性和可行性。他们在*EGFR*突变型NSCLC患者TKIs耐药后出现*MET*过表达（免疫组化IHC2+或IHC3+）和/或*MET*扩增的患者中研究了特泊替尼和吉非替尼的联合应用。在该研究的Ib期和II期Y研究中，近67%的*MET*扩增患者在接受双TKIs联合方案治疗时获得了客观缓解，并且看到这种组合是安全且可耐受的。在II期研究中共纳入55例患者，其中包括*MET*过表达（IHC3+）患者34例，*MET*扩增患者19例。在*MET*过表达（IHC3+）患者队列中：特泊替尼+吉非替尼组的mPFS为8.3个月，化疗组为4.4个月（HR=0.35, 90%CI：0.17-0.74），mOS为37.3个月，化疗组为17.9个月（HR=0.33, 90%CI: 0.14-0.76），在*MET*扩增患者队列中：特泊替尼+吉非替尼组的mPFS为16.6个月，化疗组为4.2个月（HR=0.13, 90%CI: 0.04-0.43）；特泊替尼+吉非替尼组mOS为37.3个月，化疗组为13.1个月（HR=0.08, 90%CI: 0.01-0.51），特泊替尼+吉非替尼组mOS为37.3个月，化疗组为13.1个月（HR=0.08, 90%CI: 0.01-0.51）。

尽管INSIGHT研究是一个相对小型的临床试验（MET 3+，*n*=34；*MET*扩增，*n*=19），且提前终止，但它确实阐明了在获得性EGFR抑制耐药的MET IHC 3+或*MET*扩增人群中，联合治疗相对于化疗体现出来的优势。

#### 3.2.2 特泊替尼+奥希替尼

INSIGHT 2研究^[54]^是一项开放标签、双臂II期临床研究，旨在评估特泊替尼+奥希替尼治疗含*EGFR*突变、*MET*扩增并对既往一线奥希替尼获得性耐药的晚期或转移性NSCLC患者的疗效。研究分为特泊替尼+奥希替尼组和特泊替尼单药组，主要研究终点是IRC评估的联合治疗组的ORR。2022年的ESMO大会上，研究者首次公布了INSIGHT2研究的初步结果。疗效方面，在接受特泊替尼+奥希替尼治疗并随访至少9个月的队列中，经FISH（MET GCN≥5和/或MET/CEP7≥2）检测到的22例*MET*扩增患者中，确认的ORR为54.5%（95%CI: 32.3%-75.6%），12例应答者中有6例在接受治疗。在随访至少3个月的48例患者中，ORR为45.8%（95%CI: 31.4%-60.8%）, 中位缓解持续时间DOR尚未达到。安全性方面：联合用药组23.9%的患者出现了≥3级TRAE，6.8%因TRAE停药^[54]^。两药联用的安全性特征与分别已知的安全性特征一致，整体安全良好。这组数据展示了特泊替尼+奥希替尼在一线奥希替尼进展且*MET*扩增的*EGFR*突变NSCLC患者中的积极疗效，为这部分患者提供了一种去化疗的精准治疗选择。

#### 3.2.3 赛沃替尼+奥希替尼

ORCHARD研究^[55]^是一项II期临床研究，旨在明确一线奥希替尼的耐药机制，并确定最佳的进展后治疗方案。2021年，ESMO公布了“奥希替尼+赛沃替尼”治疗*EGFR*突变型晚期NSCLC伴*MET*变异组的疗效数据：数据截止时，在20例患者中，17例可评估患者确认的ORR为41%（*n*=7, 95%CI: 25-59）。从这个小样本临床研究中，我们可以初步看到奥希替尼+赛沃替尼在治疗奥希替尼一线耐药后*MET*变异的患者中显示出的活性。

TATTON研究^[56]^是一项开放标签、多中心的Ib期临床研究，在经EGFR-TKIs治疗后耐药的*EGFR*突变NSCLC的人群中进行随机分组。探索人群中包括以不同的给药方式，以保证第一、二、三代EGFR-TKIs耐药后出现的耐药谱的全覆盖，探索了赛沃替尼与奥西替尼组合方案的疗效与安全性。在本试验中：整体*EGFR*突变NSCLC患者既往接受第一、二、三代EGFR-TKIs后耐药并有*MET*扩增的证据（FISH的检测方法定义为：在超过50个细胞平均评分≥5个MET拷贝数）的人群中：ORR为44%（22%-69%）。经第三代EGFR-TKIs治疗后进展的患者中有30%显示出客观缓解。本试验的扩展队列包括先前未经第三代EGFR-TKIs治疗的*EGFR*突变且T790M阴性的NSCLC患者，在23例患者中观察到64%的客观缓解^[14]^。据TATTON研究结果可知：对于*EGFR*突变的NSCLC患者使用任何第一、二、三代EGFR-TKIs耐药以后，采用这种TKIs联合模式均可让患者获得满意的ORR、PFS、DOR，并且安全性良好。

#### 3.2.4 卡马替尼/赛沃替尼+吉非替尼

一个Ib期/II期单臂临床试验^[19]^结果显示：经EGFR-TKIs治疗后进展的*EGFR*突变NSCLC患者中，卡马替尼+吉非替尼联合方案在*MET*基因调节异常的患者中表现出活性，特别是在*MET*扩增中，47%的肿瘤患者基因拷贝数GCN > 6和32%的*MET*过表达（IHC3+）患者实现了客观缓解。另一项探索赛沃替尼+吉非替尼的Ib期临床研究^[57]^中，有高达52%的*EGFR*突变NSCLC患者合并了*MET*扩增[扩增定义为*MET*基因GCN与7号染色体着丝粒比值（MET/CEP7 ratio）≥2或GCN≥5]，这些患者在之前的EGFR-TKIs治疗后复发，后续也获得了客观缓解。

#### 3.2.5 其他药物

非拉妥组单抗是一种人源化IgG1抗HGF的单克隆抗体。一项随机II期临床研究^[58]^评估了吉非替尼联合或不联合非拉妥组单抗治疗*EGFR*突变阳性NSCLC患者的疗效。在*EGFR*突变且*MET*高表达的亚组中，接受联合组治疗的患者41%对治疗有反应，而接受吉非替尼单药治疗的患者有22%对治疗有反应，中位PFS分别为11个月和5.5个月。然而，在整个人群中，联合治疗与吉非替尼单药治疗相比，没有显著改善疗效：ORR（40% *vs* 38%）、PFS（5.6个月 *vs* 4.7个月）和OS（24.7个月 *vs* 21.8个月），均无统计学差异。

## 4 小结与展望

十年前，合并*MET*基因突变的肿瘤在临床中被认为是好复发转移、“无药可医”、预后极差的疾病。而今，在*MET*外显子14跳跃突变患者中，多种MET选择性TKIs（卡马替尼、特泊替尼、赛沃替尼等）让我们看到了确切的疗效，成为治疗的新标准。*EGFR*突变型NSCLC患者EGFR-TKIs耐药后，当合并*MET*扩增时，通过双重通路抑制的双靶联合方案（奥西替尼+赛沃替尼，特泊替尼+吉非替尼等）更是一定程度上化解了EGFR-TKIs的耐药，成为耐药后合并*MET*扩增的有效解决方案。然而，这些证据大多基于I期和II期研究，后续尚需更多的大规模III期研究来进一步证实这些治疗策略的有效性和安全性。在免疫治疗中，特别是ICIs治疗中，近年在EGFR/ALK野生型晚期NSCLC的治疗中获得了有目共睹的成功，但在*MET*突变的患者人群中的疗效并没有确切的定论。这类ICIs相关的临床研究偏少且样本量不大，大部分研究以阴性结果为主，且在与MET-TKIs联合的研究中报告存在严重的不良反应，但随着ICIs研究的进一步深入，相信未来值得期待。*MET*基因变异在单克隆抗体、双特异性抗体及抗体偶联药物等新药中的探索大部分也为I期和II期研究，且有部分研究正在进行中，但其中已有部分研究结果让我们看到了曙光。

在期盼未来的同时，也必须面对挑战：①MET-TKIs也像EGFR/ALK-TKIs一样会出现耐药，其耐药机制复杂、后续治疗策略匮乏，这些都是需要我们思考和解决的问题；②面对不同*MET*变异形式的检测时，当前的检测手段仍存在不同程度的局限性，更加可靠有效的检测手段亟待走向临床；③在临床中进行*MET*变异的阳性判断或进行潜在可获益人群划分时，可能需要更加灵活合理的标准作为参考，需要更多更大规模的临床研究提供可靠的循证医学证据，让抗*MET*变异治疗更加精准高效。

随着相关检测技术的日益完善、新药研发及临床研究的不断拓展，MET通路的相关研究的逐步加深，NSCLC患者MET靶点领域迎来了新突破、新进展，同时也为本领域中其他癌种的研究提供了参考。
